# Effects of a combined water and sanitation intervention on biomarkers of child environmental enteric dysfunction and associations with height-for-age z-score: A matched cohort study in rural Odisha, India

**DOI:** 10.1371/journal.pntd.0009198

**Published:** 2021-03-08

**Authors:** Sheela S. Sinharoy, Heather E. Reese, Ira Praharaj, Howard H. Chang, Thomas Clasen

**Affiliations:** 1 Hubert Department of Global Health, Rollins School of Public Health, Emory University, Atlanta, Georgia, United States of America; 2 Gangarosa Department of Environmental Health, Rollins School of Public Health, Emory University, Atlanta, Georgia, United States of America; 3 Wellcome Trust Research Laboratory, Division of Gastrointestinal Sciences, Christian Medical College, Vellore, India; 4 Indian Council of Medical Research, New Delhi, India; 5 Department of Biostatistics and Bioinformatics, Rollins School of Public Health, Emory University, Atlanta, Georgia, United States of America; University of Florida, UNITED STATES

## Abstract

Poor water, sanitation and hygiene (WaSH) conditions are hypothesized to contribute to environmental enteric dysfunction (EED), a subclinical condition that may be associated with chronic undernutrition and impaired linear growth. We evaluated the effect of a combined water and sanitation intervention on biomarkers of EED, and then assessed associations of biomarkers of EED with height-for-age z-scores (HAZ), in children under five. We conducted a sub-study within a matched cohort study of a household-level water and sanitation infrastructure intervention in rural Odisha, India, in which we had observed an effect of the intervention on HAZ. We collected stool samples (N = 471) and anthropometry data (N = 209) for children under age 5. We analyzed stool samples for three biomarkers of EED: myeloperoxidase (MPO), neopterin (NEO), and α1-anti-trypsin (AAT). We used linear mixed models to estimate associations between the intervention and each biomarker of EED and between each biomarker and HAZ. The intervention was inversely associated with AAT (-0.25 log μg/ml, p = 0.025), suggesting a protective effect on EED, but was not associated with MPO or NEO. We observed an inverse association between MPO and HAZ (-0.031 per 1000 ng/ml MPO, p = 0.0090) but no association between either NEO or AAT and HAZ. Our results contribute evidence that a transformative WaSH infrastructure intervention may reduce intestinal permeability, but not intestinal inflammation and immune activation, in young children. Our study also adds to observational evidence of associations between intestinal inflammation and nutritional status, as measured by HAZ, in young children.

**Trial Registration:** ClinicalTrials.gov (NCT02441699).

## Author summary

Intestinal dysfunction due to mucosal inflammation, known as environmental enteric dysfunction, has been hypothesized to contribute to child undernutrition in low- and middle-income countries. Poor water and sanitation conditions are thought to be an underlying cause of both environmental enteric dysfunction and child undernutrition. To examine these relationships, we conducted a sub-study within a larger matched cohort study of a household-level water and sanitation intervention in rural Odisha, India. We collected stool samples from children under age five years and analyzed them for three biomarkers of environmental enteric dysfunction: myeloperoxidase, neopterin, and α1-anti-trypsin. We also assessed the same children’s nutritional status by measuring their height and calculating their height-for-age z-score. We observed a protective effect of the water and sanitation infrastructure intervention on α1-anti-trypsin. We also observed an association between myeloperoxidase and child height-for-age z-score. Our results contribute evidence that a transformative water and sanitation infrastructure intervention may reduce environmental enteric dysfunction in young children. Our study also adds to observational evidence that intestinal inflammation may be related to chronic undernutrition in young children, though the exact nature and relative importance of that relationship remains unclear.

## Introduction

Environmental enteric dysfunction (EED) is a subclinical disorder of the small intestine characterized by villous atrophy, crypt hyperplasia, decreased gut barrier function and increased permeability. EED is prevalent in individuals living in settings with poor water, sanitation, and hygiene (WaSH) conditions, especially in low- and middle-income countries (LMICs). Potential causes of EED include exposure to enteric pathogens (e.g. *Campylobacter*, *Escherichia coli*, and *Giardia*), mycotoxin exposure, and illness, especially diarrhea.[1,2]

EED has been hypothesized to be an important cause of child linear growth faltering, which in turn is a marker of future adverse outcomes including poor child development, lower schooling attainment, and reduced work capacity, productivity, and earnings in adulthood.[3,4] The effect of EED on child linear growth may operate through several mechanisms.[3,5] First, villous atrophy leads to reduced epithelial surface area and nutrient malabsorption and in turn to poor nutritional status and impaired growth.[5] At the same time, impaired gut barrier function and increased permeability allow ingested microbes to pass into the blood stream, leading to continual low-level immune activation.[5] The resulting intestinal and systemic inflammation leads to increased nutrient requirements, which, if not met, can also result in poor nutritional status and impaired growth.[5] Systemic inflammation can also lead to impaired linear growth, as inflammatory cytokines can suppress longitudinal bone growth by suppressing insulin-like growth factor 1 (IGF1).[5,6] Finally, disturbances related to EED may lead to reduced appetite and changes in the gut microbiome.[7]

Relationships between WaSH interventions, biomarkers of EED, and linear growth remain unclear. Among trials that have assessed the impact of WaSH interventions on child linear growth, a majority have observed no effect, and few have measured biomarkers of EED. To our knowledge, only two studies have published results on the effects of a WaSH intervention on both child HAZ and biomarkers of EED. The WaSH Benefits (WaSH-B) trial in rural Bangladesh observed no impact on child HAZ and no impact at a majority of time points on biomarkers of EED.[8] The Sanitation Hygiene Infant Nutrition Efficacy (SHINE) trial in rural Zimbabwe also observed no impact on child HAZ and no consistent effect on biomarkers of EED.[9] Questions remain, therefore, about the potential of WaSH interventions to impact EED and child linear growth as well as about the utility of commonly used biomarkers of EED.[10]

The objective of our study was two-fold. First, we aimed to evaluate the effect of a combined water and sanitation intervention in rural Odisha, India on three biomarkers of EED in children under five. In addition, we had previously observed evidence of an effect of the intervention on child nutritional status as measured by length/height-for-age z-score (LAZ/HAZ).[11] Thus, our second objective was to explore whether this effect may have operated through EED by assessing the associations of EED biomarkers with child LAZ/HAZ.

## Methods

### Ethics statement

The protocol for this study was reviewed by the ethics committees of the London School of Hygiene and Tropical Medicine, London, U.K. (No. 9071) and the Kalinga Institute of Medical Sciences of KIIT University, Bhubaneswar, India (KIMS/KIIT/IEC/053/2015). Anonymized data were provided to Emory University, Atlanta, U.S. under a data transfer agreement and analysis was approved by the Emory University IRB (IRB00079717). This study was registered at ClinicalTrials.gov (NCT02441699). The male and/or female household head provided written informed consent for the household.

### Study design

The EED sub-study was nested within an evaluation of the Movement and Action Network for the Transformation of Rural Areas (MANTRA) program. MANTRA was implemented by Gram Vikas, a non-governmental organization based in Odisha, India. The MANTRA intervention consisted of household-level dual-pit pour-flush toilets, bathing areas, and piped water connections to the toilet, bathing area, and kitchen. MANTRA required full community participation, such that access to the piped water system depended on all households completing toilet construction. The evaluation study took place in Ganjam and Gajapati districts of Odisha and involved a matched-cohort design with 45 randomly selected intervention villages matched to 45 control villages. In all intervention villages, the intervention began between 2003–2006 and ended by 2010.[11] Therefore, all children under age five in the current study were born post-intervention, allowing for the evaluation of longer-term impacts of the MANTRA intervention on child health outcomes.[11,12] Details of the design and methodology of the evaluation study are described elsewhere.[12]

### Participants and procedures

Data collection took place in four rounds, each lasting approximately four months, from June 2015-October 2016. Data collection methods have been reported elsewhere.[12] Briefly, households with a child under age five at any time during the study period were eligible, up to a limit of 40 households per village. In villages with more than 40 eligible households, 40 were randomly selected. Sample size calculations for the evaluation study were based on the primary outcome, diarrheal disease, and have been described elsewhere.[12] The male and/or female household head provided written informed consent for the household.

Data on individual demographics of all household members as well as household characteristics were collected as part of the evaluation study in round one (June-September 2015). For the EED sub-study, field workers requested stool samples in round two (October 2015-January 2016) and round four (July-October 2016) from all household members in a randomly selected subset of 500 households.[12] At the time of study design, to our knowledge, no studies had evaluated the effect of a WaSH intervention on biomarkers of EED, meaning that we lacked data for comprehensive sample size calculations. Therefore, we aimed for a minimum sample size of 200 children.[12] Field workers returned to households up to two consecutive days to collect stool samples; after two return visits, households that did not provide stool samples were recorded as refusals. Samples were collected without fixative.

Stool samples were processed (including noting the consistency of the sample) and were stored at -20°C in the field for at most one week and then at -80°C until analysis. Fecal AAT (μg/g) (Biovendor, Candler, NC), MPO (ng/mL) (Alpco, Salem, NH), and NEO (nmol/L) (GenWay Biotech, San Diego, CA) were analyzed at Christian Medical College (Vellore, India) using commercial kits following manufacturer’s instructions. Samples for which fecal biomarker readings exceeded the upper range of the assay were further diluted and the tests repeated. Concentrations obtained from the tests were then multiplied with the dilution factors to arrive at the final concentrations to be used for analysis. Stool samples were excluded if diarrhea was reported in the 7 days prior to stool collection. [13] The data on biomarkers of EED come from only one time point per child. Therefore, the data are treated as one cross-sectional sample, collected over a one-year period.

Anthropometry was measured using standard procedures for children <24 months in round two (October 2015-January 2016) and again for all children <60 months in round 4 (July-October 2016). Field workers measured recumbent length for children <24 months or standing height for children ≥24 months old. Length and height were measured in duplicate. If the two measurements differed by more than 0.7 cm, data collectors were required to collect a third length or height measurement on the spot. We calculated the average of the measurements for the analysis.

### Outcomes

Pre-specified outcome measures were fecal MPO, AAT, and NEO values for children under age five. An additional outcome of interest was LAZ/HAZ for children under age five, calculated using World Health Organization (WHO) growth standards.[14]

At the household level, we collected data on drinking water source, type of sanitation facility, and child feces disposal practices, all measured using a standard module from WHO/UNICEF. We calculated household sanitation use as the proportion of household members who reported usually using an improved toilet for defecation (>5 years old) or for child feces disposal (<5 years old), out of the total number of members in each household. We collected data on demographics such as caregiver schooling as well as child age and sex and validated children’s birth dates through immunization cards when possible.

The main treatment variable was the intervention status of the village where the child resided.

### Statistical analysis

We calculated descriptive and summary statistics for each biomarker of EED, including Pearson correlations of concentrations of each biomarker.

**Fig 1:**
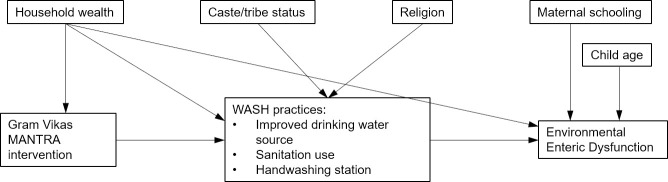
Directed acyclic graph of hypothesized relationships between MANTRA and EED.

We used linear mixed models to estimate the association between the intervention and each biomarker of EED, with a random intercept for village. We developed a directed acyclic graph (DAG) and identified potential confounders as any variables that were associated with both the exposure (the MANTRA intervention) and the outcome (EED) and not on the causal path between the exposure and the outcome in the DAG (Fig 1). We included child age because it has been observed to be associated with the concentration of biomarkers of EED, particularly in the first two years of life.[15] We also included maternal schooling as a proxy for maternal health and nutritional status, which has been theorized as an underlying factor in EED.[16]

In these models, the only variable included as an *a priori* confounder based on the DAG was the household wealth index, which was previously constructed and included 15 variables.[11] The matching process had indicated that intervention and control villages were balanced on pre-intervention household income and assets, using government data sources.[12] However, we observed a post-intervention difference between intervention and control households using our household wealth index.[11] We could not rule out the possibility that household wealth may have differed prior to the intervention by this measure; therefore we included household wealth as a potential predictor of participation in the intervention. We theorized that household wealth may also predict concentrations of biomarkers of EED through cleanliness of the overall household environment to which children are exposed.[17] Household wealth was the only socio-demographic variable for which we observed a post-intervention difference between intervention and control households, so was the only variable for which we had any evidence of potential confounding. In final models, we also included child age and maternal years of completed schooling, which in the DAG were predictors of the outcome only. We included these to decrease the amount of unexplained variability in the data and increase power to estimate an intervention effect.

Biomarker distributions and residuals from regression models using raw (un-transformed) biomarker variables exhibited substantial skewness based on statistical and graphical checks, including the Shapiro-Wilk test and Q-Q plots. We therefore log-transformed (using the natural logarithm) all three biomarker variables for these models.

We also used linear mixed models to estimate cross-sectional associations between each biomarker of EED and LAZ/HAZ, with a random intercept for village. We only included children in this analysis who had anthropometry and EED biomarker data from the same time point. Given that anthropometry was measured only for children <24 months in round two and for all children under age five in round 4 of data collection, this resulted in a smaller sample size (N = 209) than in the previous models.

**Fig 2:**
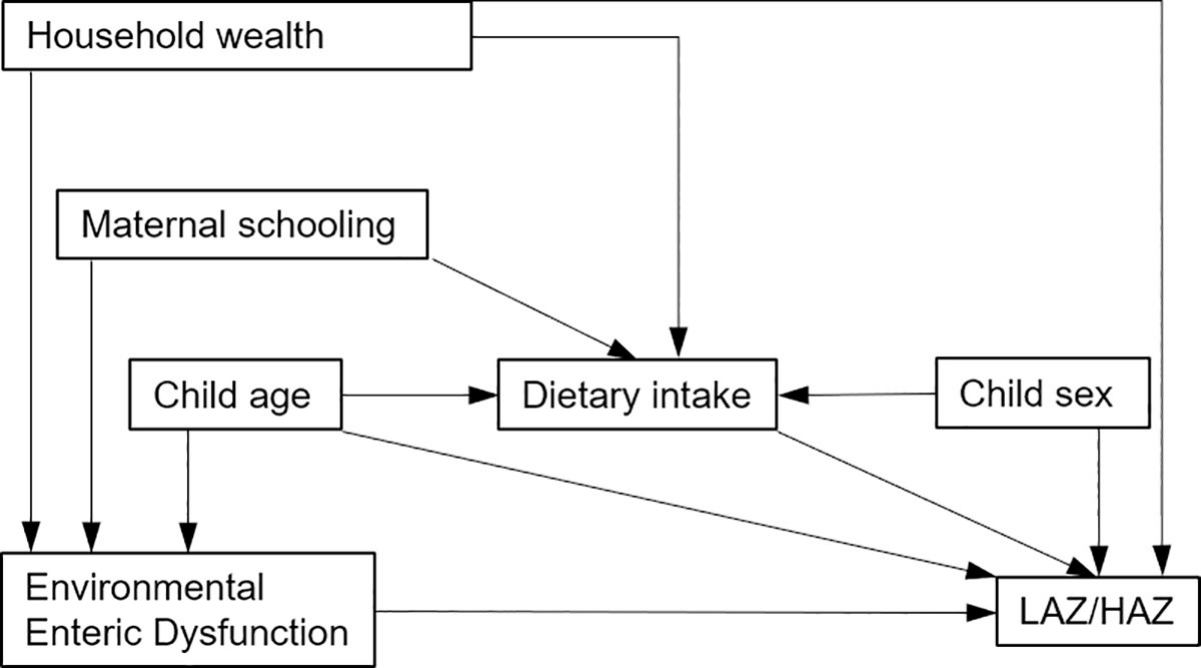
Directed acyclic graph of hypothesized relationships between EED and LAZ/HAZ.

As with the previous models, we developed a DAG, with the exposure being EED and the outcome being LAZ/HAZ (Fig 2). As in the previous DAG, we included household wealth, maternal schooling, and child age as predictors of EED. We also included household wealth as a predictor of LAZ/HAZ, both through dietary intake and independently, based on the UNICEF framework of malnutrition.[18] Child age is known to be associated with LAZ/HAZ.[19] We theorized that age would also predict dietary intake, as feeding recommendations change based on the age of an infant or young child.[20] Finally, we theorized that child sex would be associated with both dietary intake and LAZ/HAZ in this population in rural India.[21,22] Based on the DAG, we included household wealth and child age as *a priori* confounders. Also based on the DAG, we determined that child dietary intake was a collider and should be excluded from the model, as conditioning on a collider leads to a spurious association between the exposure and outcome.[23]

In contrast to the previous models, statistical and graphical checks did not indicate a need for transformation of the biomarker variables in these models. Based on these checks, we determined that the associations of the biomarker variables with LAZ/HAZ were better modeled on the raw (un-transformed) scale. However, to aid in interpretation of results, we present regression coefficients per 1000 units of the biomarker. All analyses were completed in Stata version 14.

## Results

### Characteristics of the study population

Study staff collected stool samples from 471 children under age five. These children came from 406 households, representing 86% of the 500 households that were randomly selected for the EED sub-study.

Table 1 shows descriptive statistics for children and households included in the EED sub-study. Among children who provided stool samples, their mean (± SD) LAZ/HAZ was -1.52 (± 1.35) and mean (± SD) age was 29.7 (± 14.2) months at the time of stool collection. Children were balanced across arms in terms of their age in months and sex. These characteristics were also similar to those from the overall evaluation study (Table A in S1 Text).

**Table 1:** Sociodemographic characteristics of the study population at the time of stool collection.

	Control (N = 221)	Intervention (N = 250)	p-value
Age in months, mean (SD)	29.1 (13.9)	30.1 (14.5)	0.37
Female sex (%)	46.2	51.8	0.22
LAZ/HAZ, Mean (SD) ^1^	-1.73 (1.32)	-1.33 (1.36)	0.034
Caregiver years of schooling, mean (SD)	4.3 (4.1)	5.5 (4.2)	0.031
Improved drinking water source (%)	74.7	93.6	<0.001
Handwashing station with water and soap/ash (%)	70.0	90.2	<0.001
Improved sanitation use (%)	15.7	58.5	<0.001
Wealth quintile (%)			<0.001
1 (Poorest)	23.1	14.4	
2	17.7	12.4	
3	23.5	18.8	
4	18.6	24.8	
5 (Richest)	17.2	29.6	

^1^ Sample size for LAZ/HAZ: Control = 98; Intervention = 111

As in the larger evaluation study, we observed that children participating in the EED sub-study who lived in intervention communities had higher LAZ/HAZ than those in control communities. Households in intervention communities also had higher reported percentages of improved drinking water sources and of household members using an improved sanitation facility, as well as higher observed percentages of handwashing stations with water and soap/ash. In addition, in intervention communities, mothers had more years of schooling on average, and households had higher scores on the wealth index. Household and individual characteristics were similar to the overall trial, except for the imbalance in maternal years of schooling, which was not observed in the larger trial.

### Intervention effects on biomarkers of EED

Tables 2 and 3 show summary statistics of absolute concentrations for the three EED biomarkers in the cross-sectional sample of all children participating in the EED sub-study and by intervention arm, respectively. The median values for the biomarkers in the full sample were 332.0 μg/ml AAT, 874.9 ng/ml MPO, and 1379.0 nmol/L NEO (Table 2). Among children in the intervention arm, median values are lower for AAT and higher for both MPO and NEO (Table 3). Pairwise correlations between concentrations of MPO, NEO, and AAT were low, with the highest correlation (ρ = 0.41) observed between MPO and AAT (Table 4). Boxplots of biomarker concentrations by study arm, as well as by age in months and by study arm, and correlation matrices by study arm are presented in the supplemental materials (Fig A-F in S2 Text and Tables A and B in S3 Text).

**Table 2:** Summary measures of absolute concentration of fecal EED biomarkers (n = 471).

	AAT (ug/ml)	MPO (ng/ml)	NEO (nmol/L)
Median	332.0	874.9	1379.0
25^th^ percentile	164.1	528.0	483.3
75^th^ percentile	595.4	1731.6	2626.8
Inter-quartile range	431.3	1203.6	2143.5

**Table 3:** Summary measures of absolute concentration of fecal EED biomarkers by intervention arm (n = 471).

	AAT (ug/ml)		MPO (ng/ml)		NEO (nmol/L)	
	Intervention	Control	Intervention	Control	Intervention	Control
Median	274.9	355.2	918.2	812.8	1466.035	1265.06
25^th^ percentile	130.4	207.6	577.2	495.4	649.31	420.91
75^th^ percentile	546.6	620.8	1930.3	1477.2	2832.18	2411.7

**Table 4:** Correlation matrix for concentrations of MPO, NEO, and AAT in stool (N = 471).

	MPO	NEO	AAT
MPO	1.00		
NEO	0.23	1.00	
AAT	0.41	0.32	1.00

Results of linear mixed models of each of the three fecal biomarkers on intervention arm (Table 5) indicate that the intervention was inversely associated with AAT (-0.25 log μg/ml, 95% CI: -0.48, -0.032, p = 0.025). The exponentiated value of the regression coefficient of -0.25 is 0.78, indicating that the AAT concentration among children under age five living in intervention communities is expected to be 22% lower than for those in control communities. MPO was not different between the intervention and control arms (0.12 log ng/ml, 95% CI: -0.18, 0.43, p = 0.43), nor was NEO (0.18 log nmol/L, 95% CI: -0.068, 0.44, p = 0.15). Results from fully unadjusted models are presented in Table A in S4 Text.

**Table 5:** Parameter estimates from mixed-effects linear regression models of each of the three fecal biomarkers on intervention arm (N = 471).

	Model 1		Model 2	
Biomarker	Coefficient (95% CI)	p-value	Coefficient (95% CI)	p-value
log MPO (ng/ml)	0.10 (-0.21, 0.41)	0.52	0.12 (-0.18, 0.43)	0.43
log NEO (nmol/L)	0.16 (-0.098, 0.42)	0.22	0.18 (-0.068, 0.44)	0.15
log AAT (ug/ml)	-0.25 (-0.47, -0.02)	0.031	-0.25 (-0.48, -0.032)	0.025

Model 1: Adjusted only for household wealth and village-level clustering

Model 2: Adjusted for household wealth, maternal schooling in years, child age in months, and village-level clustering

### Associations between biomarkers of EED and LAZ/HAZ

Results of linear mixed models of LAZ/HAZ on each of the three fecal biomarkers indicate that MPO was inversely associated with LAZ/HAZ (LAZ/HAZ -0.031 per 1000 ng/ml MPO, 95% CI: -0.054, -0.0076, p = 0.0090). We observed no association between LAZ/HAZ and either NEO (LAZ/HAZ +0.034 per 1000 nmol/L of NEO, 95% CI: -0.060, 0.13, p = 0.48) or AAT (LAZ/HAZ -0.14 per 1000 ug/ml of AAT, 95% CI: -0.42, 0.15, p = 0.35) (Table 6).

**Table 6:** Parameter estimates from mixed-effects linear regression models of LAZ/HAZ on each of the three fecal biomarkers (N = 209).

Biomarker	Coefficient (95% CI)	p-value
MPO (1000 ng/ml)	-0.031 (-0.054, -0.0076)	0.0090
NEO (1000 nmol/L)	0.034 (-0.060, 0.13)	0.48
AAT (1000 ug/ml)	-0.14 (-0.42, 0.15)	0.35

Note: Model adjusted for household wealth, child age in months, and village-level clustering

## Discussion

We observed evidence of a protective effect of the MANTRA intervention on AAT but not on MPO or NEO. AAT is primarily a marker of intestinal permeability, while MPO and NEO are markers of intestinal inflammation and TH1 immune activation respectively.[10] Therefore, lower AAT levels among children living in intervention villages suggest that these children have better gut function, in the form of tight junctions that allow fewer molecules to pass into the gut lumen. However, the lack of an effect on MPO or NEO suggests that this difference in intestinal permeability may not be enough to reduce intestinal inflammation and immune activation.

In our larger evaluation study, we observed an effect of the Gram Vikas MANTRA intervention on child LAZ/HAZ but no effect of the intervention on diarrhea, suggesting that the effect of the intervention on LAZ/HAZ did not operate through diarrhea.[11] The results of this sub-study further suggest that the effect of the intervention on LAZ/HAZ did not operate through biomarkers of EED. We observed evidence of an effect of the MANTRA intervention on AAT, but no association between AAT and LAZ/HAZ. We also observed an association between child LAZ/HAZ and MPO, but no effect of the intervention on MPO. Given the exploratory nature of this sub-study and that data were lacking for formal sample size calculations, we may have lacked sufficient statistical power to detect additional associations. However, in our analyses, no direct pathway existed from the MANTRA intervention to LAZ/HAZ through any of the three EED biomarkers. The key biological mechanisms mediating the effect of the MANTRA intervention on child LAZ/HAZ remain unclear.

The observed association between child LAZ/HAZ and MPO is consistent with previous studies of EED biomarkers and child linear growth. While studies have observed mixed results, MPO has been the biomarker most frequently associated with HAZ, followed by AAT, especially in younger ages.[10,24–26] Our study contributes to this body of evidence of associations between intestinal inflammation and LAZ/HAZ in young children. However, as in other studies, the small effect size suggests that EED may be a factor in linear growth faltering but is likely not the major cause.[2]

Optimal values for AAT, MPO, and NEO among children under five remain unclear. Other studies have used values reported from studies of older adolescents and adults in high-income countries as standards. These standards are <0.27 mg/g for AAT, <2000 ng/g for MPO, and <70 nmol/L.[13,26,27] Our median value for AAT (0.33 mg/g) was slightly higher than the standard of <0.27 mg/g, for MPO (874.88 ng/mL) was substantially lower than the standard of <2000 ng/g, and for NEO (1379.02 nmol/L) was higher than the standard of 70 nmol/L. However, the interpretation of these differences is not clear, given that the standards are based on limited data, from non-representative samples of different ages than the individuals in our study, and are from studies of medical conditions such as inflammatory bowel disease rather than EED.

The median biomarker values from our sub-study population also differed substantially from those observed in the Malnutrition and Enteric Diseases (MAL-ED) study, which assessed AAT, MPO, and NEO in stool samples from young children in India. The median values from the Indian sample in MAL-ED were 0.59 mg/g for AAT, 14,574.97 ng/mL for MPO, and 2009.31 nmol/L for NEO, all of which were higher than values observed in our study.[13] Of note, the samples from MAL-ED and our study were assessed in the same lab, using the same assays and methods.[13] However, the ages of the target population were different, as the MAL-ED data came from infants at 3, 6, and 9 months of age while our data include children up to age five. Research suggests that concentrations of each of these biomarkers may decrease with child age, with levels stabilizing after 2 years of age.[2,15]

The median values for AAT, MPO, and NEO observed in our sub-study population were higher than standards from high-income countries but lower than those observed among children in India in the MAL-ED study. Importantly, this difference cannot be attributed solely to the MANTRA intervention, as even within the control group of our sub-study, median values of all three biomarkers were lower than those observed in the MAL-ED study. It is also worth noting, when comparing biomarker values from children in our sub-study to standards from high-income countries, that the MANTRA intervention does not address potential sources of contamination such as soil, animal feces, or poor food hygiene, which may represent important transmission pathways for young children.[28,29] Addressing these may be necessary to achieve more comprehensive improvements to environmental conditions.

Studies thus far have documented inconsistent effects of WaSH infrastructure interventions on biomarkers of EED in children. The WASH-B study in rural Bangladesh assessed biomarkers of EED in children at median ages 3, 14, and 28 months. Results suggested that the WaSH intervention (which included construction or improvement of latrines, drinking water treatment, and handwashing stations) had limited effects on EED. In the WaSH intervention arm compared to controls, concentrations of AAT did not differ at any age; concentrations of MPO did not differ at 3 and 14 months but were unexpectedly higher at age 28 months; and concentrations of NEO were lower at 3 months but did not differ at 14 or 28 months.[8] Similarly, the SHINE trial in rural Zimbabwe assessed biomarkers of EED at ages 1, 3, 6, 12, and 18 months. Results suggested no consistent effect of the WaSH intervention (in which the infrastructure component included a ventilated improved pit latrine and two handwashing stations) on any biomarkers.[9] These results contrast with the results from our study, which observed evidence of an effect on AAT and no effect on MPO or NEO.

The difference in EED results between the WaSH-B and SHINE trials and our study may relate to the differences in the interventions. The WaSH-B and SHINE trial interventions involved basic sanitation services and no increase in water access, whereas the Gram Vikas MANTRA intervention involved safely managed sanitation services combined with increased access to basic water services. The WaSH-B and SHINE trials also did not aim to achieve community-level coverage, whereas the Gram Vikas MANTRA intervention targeted all households in a community. Thus, the Gram Vikas MANTRA intervention is more in line with a comprehensive or ‘transformative’ WaSH approach, which may explain the differences in observed effects on both LAZ/HAZ and biomarkers of EED.

Our study has several strengths and limitations. A strength of the study is the use of a matched cohort design to assess the long-term impacts of a previously completed intervention on biomarkers of EED. The comparison group was identified through a rigorous matching process, resulting in balance across observed socio-demographic characteristics between intervention and comparison arms at the village level.[12] However, the study design also presents a limitation in that it did not involve random assignment of the intervention, resulting in the possibility that villages were not balanced on unobserved characteristics. In addition, the analysis of associations between biomarkers of EED and LAZ/HAZ (unlike the analysis of associations between the MANTRA intervention and biomarkers of EED) was cross-sectional and therefore cannot be used for causal inference. For all analyses, data were lacking for comprehensive sample size calculations, so statistical power may have been insufficient.

The intervention villages included in our study were randomly selected from a list of villages where Gram Vikas had implemented the MANTRA program. Therefore, our study results are generalizable to villages that have received the MANTRA intervention. Gram Vikas has completed the intervention in more than 1000 villages across Odisha, reaching over 350,000 people.[30] However, it is important to note that the results cannot be generalized to other types of WaSH interventions, especially those that provide only basic services and do not aim to achieve community-level coverage.

## Conclusion

Our results contribute evidence that a household-level water and sanitation infrastructure intervention may reduce intestinal permeability, and that intestinal inflammation is associated with growth faltering, in children under five. More research is needed to: (a) establish optimal values for AAT, MPO, and NEO among children under five in LMICs; (b) identify interventions that work to reduce the prevalence of EED in young children; and (c) elucidate pathways through which WaSH infrastructure interventions may impact child linear growth. Transformative WASH approaches may be necessary to reverse and prevent EED and to promote linear growth in young children in low- and middle-income country settings.

## Supporting information

S1 Text Table A in S1 Text. Enrollment characteristics by intervention group within the Gram Vikas MANTRA evaluation study population and within the EED sub-study population.
(DOCX)

S2 Text Figure A in S2 Text. Boxplot of myeloperoxidase (MPO) biomarker concentration, by intervention status. **Figure B in S2 Text**. Boxplot of neopterin (NEO) biomarker concentration, by intervention status. **Figure C in S2 Text**. Boxplot of α1-anti-trypsin (AAT) biomarker concentration, by intervention status. **Figure D in S2 Text**. Boxplot of log myeloperoxidase (MPO) biomarker concentration, by age in months and by intervention status. **Figure E in S2 Text**. Boxplot of log neopterin (NEO) biomarker concentration, by age in months and by intervention status. **Figure F in S2 Text.** Boxplot of log α1-anti-trypsin (AAT) biomarker concentration, by age in months and by intervention status.
(DOCX)

S3 Text Table A in S3 Text. Pearson correlation matrix for concentrations of MPO, NEO, and AAT in stool, control arm only (N = 221). **Table B in S3 Text.** Pearson correlation matrix for concentrations of MPO, NEO, and AAT in stool, intervention arm only (N = 250).
(DOCX)

S4 Text Table A in S4 Text. Parameter estimates from mixed-effects linear regression models of each of the three fecal biomarkers on intervention arm (N = 471).
(DOCX)
