# Supplementary material for: Effects of a combined water and sanitation intervention on biomarkers of child environmental enteric dysfunction and associations with height-for-age z-score: A matched cohort study in rural Odisha, India
Source: PLoS Negl Trop Dis. 2021 Mar 8;15(3):e0009198. doi: 10.1371/journal.pntd.0009198 (PMC7971857; doi:10.1371/journal.pntd.0009198)
Supplement: S1 Text — Enrollment characteristics by intervention group within the Gram Vikas MANTRA evaluation study population and within the EED sub-study population. (DOCX) [file pntd.0009198.s001.docx]

**Supporting Information: S1 Text**

**Table A.** Enrollment characteristics by intervention group within the Gram Vikas MANTRA evaluation study population and within the EED sub-study population

|  | Gram Vikas MANTRA evaluation study | | EED sub-study | |
| --- | --- | --- | --- | --- |
| Household characteristics | Control (N=1275) | Intervention (N=1123) | Control (N=221) | Intervention (N=250) |
| Caregiver education ≥5 years (%) | 48.0 | 57.0 | 61.1 | 47.2 |
| Improved drinking water source (%) | 72.0 | 92.1 | 74.7 | 93.6 |
| Handwashing station with water and soap/ash (%) | 62.4 | 76.1 | 70.0 | 90.2 |
| Improved sanitation use (%) | 12.9 | 59.3 | 15.7 | 58.5 |
| Wealth quintile (%) |  |  |  |  |
| 1 | 25.3 | 14.9 | 23.1 | 14.4 |
| 2 | 20.0 | 19.2 | 17.7 | 12.4 |
| 3 | 20.6 | 19.4 | 23.5 | 18.8 |
| 4 | 18.0 | 22.5 | 18.6 | 24.8 |
| 5 | 15.8 | 24.1 | 17.2 | 29.6 |
| Individual characteristics | Control (N=1797) | Intervention (N=1502) | Control (N=221) | Intervention (N=250) |
| Age in months, Mean (SD) | 28.5 (17.7) | 29.4 (17.7) | 29.1 (13.9) | 30.1 (14.5) |
| Female sex (%) | 49 | 49.2 | 46.2 | 51.8 |
| LAZ/HAZ, Mean (SD) | -1.77 (1.12) | -1.48 (1.17) | -1.73 (1.32) | -1.33 (1.36) |
