# Supplementary material for: Effects of a combined water and sanitation intervention on biomarkers of child environmental enteric dysfunction and associations with height-for-age z-score: A matched cohort study in rural Odisha, India
Source: PLoS Negl Trop Dis. 2021 Mar 8;15(3):e0009198. doi: 10.1371/journal.pntd.0009198 (PMC7971857; doi:10.1371/journal.pntd.0009198)
Supplement: S2 Text — Boxplot of myeloperoxidase (MPO) biomarker concentration, by intervention status. Figure B in S2 Text. Boxplot of neopterin (NEO) biomarker concentration, by intervention status. Figure C in S2 Text. Boxplot of α1-anti-trypsin (AAT) biomarker concentration, by intervention status. Figure D in S2 Text. Boxplot of log myeloperoxidase (MPO) biomarker concentration, by age in months and by intervention status. Figure E in S2 Text. Boxplot of log neopterin (NEO) biomarker concentration, by age in months and by intervention status. Figure F in S2 Text. Boxplot of log α1-anti-trypsin (AAT) biomarker concentration, by age in months and by intervention status. (DOCX) [file pntd.0009198.s002.docx]

**Supporting Information: S2 Text**

**Figure A.** Boxplot of myeloperoxidase (MPO) biomarker concentration, by intervention status

**Figure B.** Boxplot of neopterin (NEO) biomarker concentration, by intervention status

**Figure C.** Boxplot of α1-anti-trypsin (AAT) biomarker concentration, by intervention status

**Figure D.** Boxplot of log myeloperoxidase (MPO) biomarker concentration, by age in months and by intervention status

**Figure E.** Boxplot of log neopterin (NEO) biomarker concentration, by age in months and by intervention status

**Figure F.** Boxplot of log α1-anti-trypsin (AAT) biomarker concentration, by age in months and by intervention status
