# Supplementary material for: Effects of a combined water and sanitation intervention on biomarkers of child environmental enteric dysfunction and associations with height-for-age z-score: A matched cohort study in rural Odisha, India
Source: PLoS Negl Trop Dis. 2021 Mar 8;15(3):e0009198. doi: 10.1371/journal.pntd.0009198 (PMC7971857; doi:10.1371/journal.pntd.0009198)
Supplement: S3 Text — Pearson correlation matrix for concentrations of MPO, NEO, and AAT in stool, control arm only (N = 221). Table B in S3 Text. Pearson correlation matrix for concentrations of MPO, NEO, and AAT in stool, intervention arm only (N = 250). (DOCX) [file pntd.0009198.s003.docx]

**Supporting Information: S3 Text**

**Table A.** Pearson correlation matrix for concentrations of MPO, NEO, and AAT in stool, control arm only (N=221)

|  | MPO | NEO | AAT |
| --- | --- | --- | --- |
| MPO | 1.00 |  |  |
| NEO | 0.28 | 1.00 |  |
| AAT | 0.45 | 0.33 | 1.00 |

**Table B.** Pearson correlation matrix for concentrations of MPO, NEO, and AAT in stool, intervention arm only (N=250)

|  | MPO | NEO | AAT |
| --- | --- | --- | --- |
| MPO | 1.00 |  |  |
| NEO | 0.20 | 1.00 |  |
| AAT | 0.39 | 0.33 | 1.00 |
