# Supplementary material for: Effects of a combined water and sanitation intervention on biomarkers of child environmental enteric dysfunction and associations with height-for-age z-score: A matched cohort study in rural Odisha, India
Source: PLoS Negl Trop Dis. 2021 Mar 8;15(3):e0009198. doi: 10.1371/journal.pntd.0009198 (PMC7971857; doi:10.1371/journal.pntd.0009198)
Supplement: S4 Text — Parameter estimates from mixed-effects linear regression models of each of the three fecal biomarkers on intervention arm (N = 471). (DOCX) [file pntd.0009198.s004.docx]

**Supporting Information: S4 Text**

**Table A.** Parameter estimates from mixed-effects linear regression models of each of the three fecal biomarkers on intervention arm (N=471)

|  | Model 1 | | Model 2 | |
| --- | --- | --- | --- | --- |
| Biomarker | Coefficient (95% CI) | p-value | Coefficient (95% CI) | p-value |
| MPO | -200.24 (-1309.04, 908.56) | 0.72 | -248.35 (-1328.68, 831.98) | 0.65 |
| NEO | 276.56 (-106.01, 659.12) | 0.15 | 299.11 (-67.93, 666.16) | 0.11 |
| AAT | -31.82 (-139.97, 76.33) | 0.56 | -37.46 (-142.15, 67.24) | 0.48 |
| Model 1: Adjusted only for village-level clustering | | | | |
| Model 2: Adjusted for household wealth, maternal schooling in years, child age in months, and village-level clustering | | | | |
